# It starts at home: non-economic factors influencing consumer acceptance of battery storage in Australia

**DOI:** 10.1007/s11356-024-32614-5

**Published:** 2024-02-27

**Authors:** Breda McCarthy, Hongbo Liu

**Affiliations:** 1 Department of Economics and Marketing, James Cook University (JCU), Douglas, Townsville, Queensland 4818 Australia; 2 Academy of Strategies for Innovation and Development, Anhui University, No 3, Feixi Road, Hefei, 230039 Anhui Province China

**Keywords:** Battery storage, Social–psychological drivers, Barriers, Demographics, Prosumers, Regression analysis

## Abstract

Homeowners play a critical role in the uptake of low-carbon technologies, yet little is known about the factors that underlie market acceptance of residential battery storage. This research integrates social–psychological, demographic and behavioural factors into a holistic model that predicts market acceptance. Previous research has indicated that social factors play a crucial role in the adoption of rooftop solar. Still, the influence of subjective norms on battery storage, a relatively invisible technology, has yet to be fully understood. An online survey from homeowners in Australia, a mature renewable energy market, is used to provide insights into market acceptance that are relevant to international energy markets. A two-step econometric model, using factor analysis and ordered logistic regression, was used for data analysis. The results show that subjective norms, moral emotions and an environmental self-identity are positively associated with market acceptance. Demographic factors, such as younger age and higher levels of education, predict market acceptance. Motives such as technical interest, autarky and load-shifting behaviours are also relevant. Several recommendations for policymakers and practitioners are offered to improve the acceptance of battery storage, including interventions that exploit social parameters and appeal to consumer psychology.

**Supplementary Information:**

The online version contains supplementary material available at 10.1007/s11356-024-32614-5.

## Introduction

Battery storage plays a pivotal role in the energy sector’s decarbonisation (Kittner, Lill and Kammen 2017) and is instrumental in meeting climate change mitigation targets (UNCCC 2021). Renewable energy sources, such as solar and wind, have inherently variable outputs. Their energy production is influenced by daily cycles, weather patterns and other external factors. This is where battery storage systems come into play. They can harness excess energy during peak production hours and subsequently dispense it during lulls, ensuring a consistent and dependable power flow (International Energy Agency 2021). Moreover, batteries are adept at reacting swiftly to sudden shifts in energy demand. This agility helps preserve an equilibrium between supply and demand, stabilising the grid, even amidst an increasing flow of renewable energy (Esplin and Nelson 2022). In addition, battery storage systems can function autonomously from the primary grid. This not only minimises transmission losses but also fortifies the energy system’s resilience to potential disruptions (Agnew, Smith and Dargusch 2018). In essence, battery storage helps achieve crucial energy goals of enhanced efficiency, security, reliability and sustainability in electricity provision.

There is a rich, well-established literature on residential rooftop solar adoption (Best, Burke and Nishitateno 2019; Esplin and Nelson 2022; Sommerfeld et al. 2017) and adoption behaviour has been extensively analysed—encompassing environmental concern, financial motivations and peer effects (Alipour, Irannezhad, Stewart and Sahin 2022; Schulte, Scheller, Sloot and Bruckner 2022). Environmental values, encapsulating an individual’s principal life aspirations, together with self-identity—defined as an individual’s introspective self-perception—have been identified as salient predictors of pro-environmental behaviours (Bouman, van der Werff, Perlaviciute and Steg 2021; Whitmarsh and O’Neill 2010). However, research in the context of battery storage is still rare. As noted by Agnew et al. (2018, p. 2,364), ‘the residential battery energy market is currently at an embryonic stage of development, and there exists very little market data and limited primary research regarding adoption dynamics’. As elucidated by scholars such as Barr et al. (2011), consumer behaviour exhibits a multi-dimensional nature, and motives are contingent upon specific contexts. This underscores the need for tailored, context-specific research endeavours (Best et al. 2021). A growing body of literature is focused on exploring the policy incentives that underlie consumer adoption of battery storage (Fett et al. 2021). While much of the research on battery storage gravitates towards economic determinants (Best, Li, Trück and Truong 2021), there is a growing chorus urging exploration of non-economic drivers of adoption (Esplin and Nelson 2022). As Klingler (2017) highlights, consumers are embracing this technology even when its economic feasibility remains questionable. Therefore, delving into consumers’ motives for accepting battery storage and their energy-oriented beliefs and practices is paramount.

The prevailing literature on battery storage frequently marginalises psychological variables and the intricate behavioural dynamics pertinent to battery storage adoption. For instance, research shows that people are influenced by the perceived expectations or behaviour of others, typically labelled ‘peer effects’ or ‘spatial proximity’ (Moncada, Tao, Valkering, Meinke-Hubeny and Delarue 2021; Mundaca and Samahita 2020). However, evidence of social influence on battery storage is scarce, with scholars concluding that social influence is irrelevant to battery storage since the setting is private (Alipour, Taghikhah, Irannezhad, Stewart and Sahin 2022). By engaging with this debate on social influence, this study addresses a gap in the literature. The novelty of this study lies in its focus on consumer acceptance of battery storage and the integration of multiple predictors in a holistic framework. This study focuses on Australia since it stands at the forefront of global trends with significant household uptake of solar photovoltaic (PV) systems (Best et al. 2019). This research makes a pivotal contribution to academic discourse and practice in several ways:

- 1.This study delves into the antecedents of battery storage acceptance, grounded in the theory of planned behaviour (Ajzen 1991). By scrutinising relatively underexplored concepts in the battery storage literature, our work is a pertinent addition to the ongoing scholarly debates.
- 2.This research critically evaluates consumer reactions to ‘prosumer’ and battery leasing paradigms, wherein either a household or an aggregator orchestrates consumption and storage to simultaneously benefit individuals and the broader system (Esplin and Nelson 2022). Such an investigation holds paramount importance, especially when consumer-oriented research on battery storage is scarce (Kalkbrenner 2019) and studies specific to the Australian context are, to our understanding, conspicuously absent.
- 3.The study furnishes insights for policymakers and industry stakeholders both within Australia and internationally. The overarching objective is to fine-tune strategies that bolster the growth of this market, thereby advancing global climate change mitigation endeavours.

This paper is structured as follows. ‘Theoretical model and hypotheses development’ section analyses the theoretical foundations of the study and outlines the hypotheses. The methodology is outlined in ‘Method’ section, and the results are presented in ‘Results’ section. Finally, ‘Discussion’ section discusses the findings, outlines the limitations and proposes avenues for future research.

## Theoretical model and hypotheses development

**Fig. 1:**
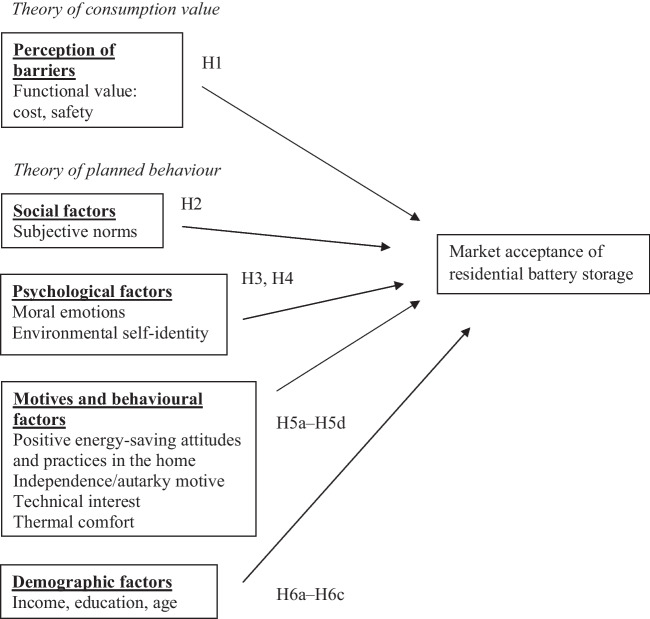
Conceptual framework: antecedents of residential battery storage acceptance

Battery storage is defined as an energy storage technology that uses chemicals to absorb and release energy on demand (Australian Renewable Energy Agency [ARENA], 2022). This study examines the impact of social–psychological, behavioural and demographic factors on the market acceptance of residential battery storage. The conceptual framework is shown in Fig. 1. While our research does not set out to craft a novel theoretical framework or extend the theory of planned behaviour (Ajzen 1991), it offers empirical insights that validate and enrich current academic paradigms. The main research questions that are addressed in this study are as follows:

- What is the impact of perceived barriers on the acceptance of battery storage?
- What is the impact of social–psychological factors (i.e. subjective norms, moral emotions, environmental self-identity) on the acceptance of battery storage?
- What is the impact of energy-related motives (i.e. independence, technical interest) and behavioural factors (i.e. thermal comfort needs, electricity saving) on the acceptance of battery storage?
- How do demographic factors (i.e. age, education, income) affect the acceptance of battery storage?

Growing attention is being paid to business models in a post-subsidy market that can offer value to energy consumers (Karami and Madlener 2022). For instance, different types of peer-to-peer trading models have been identified by scholars (Schwidtal et al. 2023), yet little is known about consumers’ attitudes towards prosumerism (Pena-Bello et al. 2022). The term ‘prosumer’ refers to a person or entity who consumes and produces energy, resulting in a two-way flow of energy (Dütschke, Galvin and Brunzema 2021; Parag and Sovacool 2016; Saleh 2018). One study shows that German households decide to become prosumers for financial reasons (Karami and Madlener 2022), environmental concerns, technical interests and independence aspirations (Hackbarth and Löbbe 2020). Furthermore, new business models, such as leasing, are expected to add value to the battery storage market, but they are currently only offered to commercial customers in the Australian market (Energy Matters 2022a). To what extent Australian consumers are willing to embrace solar trading and leasing remains unclear. Therefore, we gathered descriptive statistics to address the following practitioner-oriented question:

- What are consumers’ attitudes towards business models that incentivise people to act as prosumers, and how do prosumers differ from non-prosumers?

### Acceptance of battery storage and perceptions of barriers

Social acceptance is a prominent topic of research. While it has been skewed towards understanding resistance to technology, such as the ‘NIMBY’ (not in my back yard) concept, Devine-Right et al. (2017) argue that understanding the gamut of reactions to new energy technologies by different actors and at different scales, remains important, particularly given the lag between technological innovation and technology diffusion. The term ‘acceptance’ is not well defined in the literature, but in the Technology Acceptance Model by Davis (1985), it is defined as ‘actual system use’. However, for energy researchers, acceptance is defined more broadly and can be expressed in various forms, such as attitudes, behaviour and investment (Wüstenhagen, Wolsink and Bürer 2007). Therefore, based on the energy literature, we use the term ‘acceptance’, which equates to an interest in and support for battery storage, and not actual use.

It is necessary to explain perceived barriers to battery storage before exploring why consumers accept or fail to accept battery storage. In the broader literature on technology acceptance, numerous factors affect perceptions of technology, including perceived usefulness, ease of use, attitudes, social influence and cultural factors (Yigitcanlar et al. 2023). The theory of consumption value, developed by Sheth et al. (1991), is a marketing theory that provides insight into the motivation for consumers’ consumption behaviour. Value is a multi-dimensional concept and covers functional, social, emotional, epistemic and conditional value (Tanrikulu 2021). Functional value is determined by a product’s attributes, such as price, reliability and durability (Tanrikulu 2021). Technological limitations can erode the functional value and hamper the acceptance of innovation, such as electric vehicles (Pinto et al. 2022). High cost is a critical issue slowing the broad market penetration of residential battery storage (Alipour et al. 2022; Heymans et al. 2014). The focus of research in new energy technologies is generally on government policy mechanisms that help overcome barriers to adoption (Das and Bhat 2022). For instance, in relation to storage, scholars highlight the importance of feed-in tariffs (FiTs), which is a payment or a credit on the electricity bill, for exporting solar to the grid (Best et al. 2021). The adoption of battery storage is often framed as an investment decision; acceptable payback periods (van Groenou, Lovell and Franklin, 2018), which refer to the amount of time it takes to recover the cost of an investment, are found to be instrumental in motivating battery storage uptake.

In Australia, there are incentives for installing batteries (i.e. rebates and interest-free loans) that vary by state and territory (Energy Matters 2022b). However, for some Australian households, there is a disincentive to invest in storage. Early adoption of solar is seen as a barrier to adopting battery storage (Esplin and Nelson 2022). Households that installed solar PV before 2012 received a generous FiT of 44 cents (AUD) per kilowatt-hour. To maintain eligibility for the premium FiT, innovators and early adopters have an incentive to sell surplus electricity back to the grid and delay investing in storage to obtain the most benefit from the contract before it ends in 2028 (Alipour et al. 2022). Likewise, qualitative research in Germany revealed that further investments in renewable energy were held back as households still enjoyed a high FiT and did not want to change their solar configuration (Dütschke et al. 2021).

Safety concerns are a potential barrier to the acceptance of battery storage since lithium-ion batteries have been recalled due to explosion and fire accidents (Chen et al. 2021). Research highlights the importance of safety in driving public acceptance of batteries (Agnew and Dargusch 2017; Kalkbrenner 2019). However, many other factors could inhibit adoption, such as transient employment, people expecting to move house and the house not being physically suitable (Alipour et al. 2022). Thus, the following hypothesis is advanced:

- H1. Perceptions of barriers negatively influence acceptance of battery storage.

### Subjective norms and the theory of planned behaviour

The theory of planned behaviour was initially developed by Ajzen (1991) to represent the psychological determinants of behaviour, and it builds on the theory of reasoned action (Ajzen and Fishbein 1980). Human behaviour is said to be influenced by three key constructs: attitudes (favourable/unfavourable evaluations of the behaviour), subjective norms (perceptions of social pressure to perform the behaviour) and perceived behavioural control (perceived ability to perform the behaviour) (White et al. 2023). The theory is widely cited and has been used in a variety of domains, including sustainable housing (Judge, Warren-Myers and Paladino 2019), electric cars (Liu et al. 2020), organic food (Bósquez and Arias-Bolzmann 2021), green products (Yadav and Pathak 2017), rooftop solar adoption (Liu et al. 2020), health research (Lei, Deeprasert, Li and Wijitjamree 2022), pro-social behaviour, such as charitable donations (White et al. 2023), and pro-environmental behaviours in general (Carfora, Caso, Sparks and Conner 2017; Cook, Kerr and Moore 2002). Few studies have examined the influence of subjective norms on battery storage acceptance (Alipour et al. 2022). Considering the importance of battery storage in the decarbonisation of the energy sector, how social parameters influence acceptance remains a critical but unanswered question.

In the theory of planned behaviour, two types of norms can be distinguished. Injunctive norms are social pressures to engage in behaviour based on the perception of what other people want an individual to do, and descriptive norms are social pressures based on the observed or inferred behaviour of others. Norms are akin to social approval, which refers to the extent to which society, in general, condones engagement in a particular behaviour (Manning 2009). The literature recognises the central role of peer networks in the diffusion of renewable energy, such as observing solar panels on the roofs of other homes and being influenced by one’s neighbours (Bollinger and Gillingham 2012; Curtius, Hille, Berger, Hahnel and Wüstenhagen 2018; Lan, Cheng, Gou and Yu 2020; Wolske, Gillingham and Schultz 2020). The growing literature on electric vehicle adoption (Adnan, Nordin, Rahman and Amini 2017) reports similar insights. In other words, people learn about the benefits of solar panels through observation and imitate commonplace behaviour. Normative influence is also relevant to the adoption of electric vehicles (Sajjad, Asmi, Chu and Anwar 2020). However, the researchers assert that in contrast to visible technologies, such as rooftop solar and cars, the adopters of battery storage are less influenced by their peers. It is suggested that the lack of visibility of the battery system might reduce the role of neighbours (or descriptive norms) in the decision process compared with rooftop panels. Another explanation is that the adopters are innovators and forward-looking, and it is suggested that peer effects are more likely to operate in the earlier stages of attitudinal formation long before the intention or investment decision is made (Alipour et al. 2022). Considering the inconclusive findings in the literature, one objective of this study is to examine whether subjective (or injunctive) norms predict battery storage acceptance:

- H2. Subjective norms positively influence the acceptance of battery storage.

### Moral emotions

Norms and moral emotions are concepts that work together in explaining human behaviour. In relation to the long-established theory of the Norm Activation Model (Schwartz 1977), people are more likely to invest in battery storage (and act pro-environmentally) when they experience a strong personal norm to save energy. Moral emotions are defined as ‘the emotions that respond to moral violations or that motivate moral behaviour’ and that ‘go beyond the direct interests of the self’ (Haidt 2003, p. 852). For example, guilt is a negative emotion which grows out of communal relationships. As a negative emotion, it focuses attention on a problem and sets in motion a corrective action (Haidt 2003). In the area of energy conservation, it is proposed that if a person feels social pressure to save electricity and violates that norm, the result will be guilt, a moral emotion. Accordingly, another hypothesis is formulated:

- H3. Moral emotions, such as guilt, positively influence the acceptance of battery storage.

### Environmental self-identity

Self-identity is generally interpreted as ‘the label that people use to describe themselves’ (Cook et al. 2002, p. 559). Self-identity is found to be a distinctive predictor of intentions in Ajzen’s (1991) theory of planned behaviour (Paquin and Keating 2017). Furthermore, when people strongly identify with the reference group, the intention to perform the behaviour is strongly influenced by perceived norms (Terry, Hogg and White 1999). A pro-environmental self-identity significantly predicts a range of pro-environmental behaviours, such as waste reduction, water and domestic energy conservation, eco-shopping and eating (Whitmarsh and O’Neill 2010). An environmental self-identity is said to form a robust and stable motivational basis for climate action (Bouman et al. 2021) and has been validated cross-culturally (Dermody, Hanmer-Lloyd, Koenig-Lewis and Zhao 2015). With the above considered, the following hypothesis is proposed:

- H4. A pro-environmental self-identity positively influences battery storage acceptance.

### Motives and behavioural determinants of battery acceptance

Energy-related behaviours have been extensively studied (Thøgersen and Grønhøj, 2010; Wallis, Nachreiner and Matthies 2016). However, the interrelationships between occupant behaviour and the acceptance of battery storage have been omitted from prior research. A key question is whether the energy-related behaviour of consumers is associated with battery storage acceptance. Without solid evidence for the link between daily behaviour and battery storage acceptance, this research question will be answered in an exploratory manner. Lending some support to this approach, Dütschke et al. (2021) assert that the energy-related behaviour of households must be aligned with demand reduction goals.

Households have a critical impact on energy consumption (Gardner and Stern 2008; Umit, Poortinga, Jokinen and Pohjolainen 2019) through behaviours such as the purchase of energy services (e.g. number and efficiency of appliances) and the conservative use of appliances. The term ‘energy conservation’ encompasses a diverse set of behaviours that widely vary in terms of relative financial cost, effort and the knowledge required to implement them (Gardner and Stern 2008; Karlin et al. 2014). For example, adjusting thermostats, turning off lights when not needed and taking shorter showers are also examples of measures that can be taken to reduce energy consumption. Engaging in ‘load-shifting’ behaviour can also help households manage their electricity consumption. Load shifting refers to attempts by utilities to use pricing signals to reduce demand for electricity at critical periods, known as ‘peak demand’ (i.e. periods when electricity usage on the network is at its highest) (Wittenberg and Matthies 2016).

Motives underlying energy-related behaviours have been extensively studied. Economic considerations, including concerns about rising electricity bills, are the most cited motivations for conserving electricity (Xu, Shu, Shao and Xiang 2021) and for adopting solar PV (Sommerfeld, Buys and Vine 2017, 2017). Therefore, self-interest, not just altruistic motives, helps explain pro-environmental behaviour (Abrahamse, Steg, Gifford and Vlek 2009). Among low-income groups, bill consciousness positively predicts energy conservation intentions (Chen, Xu and Day 2017). Regarding motives for installing battery storage, it has been found that energy self-sufficiency, or not relying on the grid for electricity, is a strong driver of battery adoption (Agnew and Dargusch 2017). A recent study found that independence from the grid is a stronger motivator for intentions to install battery storage than environmental concerns (Alipour et al. 2022). Prior research shows that German households decide to become prosumers due to technical interests and independence aspirations (Hackbarth and Löbbe 2020). A common criticism of the theory of planned behaviour is that it overlooks a lack of motivation when predicting human behaviour (Lei et al. 2022); thus, by considering the motives for accepting battery storage, this study helps extend prior scholarship.

The concept of thermal comfort is important in studies of electricity conservation (Huebner, Cooper and Jones 2013; Ren and Chen 2018; Yang, Yan and Lam 2014). Thermal comfort describes what people want from energy services, for example, how warm or cool they expect their house to be (Stephenson et al. 2015). The desire for comfort is an inherent benefit of energy services, one that influences investment in ‘green’ buildings (Bond 2011; Samuelson and Biek 1991). However, research shows that it negatively affects energy conservation intentions (Chen et al. 2017). Air conditioning use is widespread in many parts of Australia due to its tropical climate. It is predicted that an increase in the intensity and duration of heat waves due to climate change (Sachindra, Ng, Muthukumaran and Perera 2016) will lead to high demand for cooling (Ren and Chen 2018). Several consecutive summers of heat waves and drought have strained people and nature in Australia. Therefore, it is logical to assume that battery storage will be desirable to consumers as a long-term home-cooling option. Batteries enable consumers to avoid consuming electricity from the grid during peak periods and still maintain comfort. Based on the literature above, it is hypothesised that certain motives, attitudes and behaviours towards electricity saving will be aligned with battery storage acceptance. The following hypotheses are advanced:

- H5a. Positive attitudes and behaviours towards electricity saving positively influence the acceptance of battery storage.
- H5b. The energy independence motive positively influences the acceptance of battery storage.
- H5c. Technical interest positively influences the acceptance of battery storage.
- H5d. A desire for thermal comfort is positively associated with the acceptance of battery storage.

### Demographics

Demographic characteristics are often studied since they help explain energy-related behaviours (Brounen, Kok and Quigley 2012; Chen et al. 2017; Wallis et al. 2016). Research findings on energy saving, energy-related investments and socio-demographics tend to be contradictory (Umit et al. 2019), but some factors emerge as influential. Income is seen as an important predictor of battery storage adoption (Brown 2022), and solar uptake has a strong middle-income effect (Bondio, Shahnazari and McHugh 2018; Jacksohn, Grösche, Rehdanz and Schröder 2019). In an Australian study, Best et al. (2021) found that households experiencing financial pressure (a measure of capital constraints) were less likely to install batteries. Previous literature on rooftop solar adoption has reported a positive correlation between income and solar installations (Nelson, Simshauser and Kelley 2011). More broadly, the affluence hypothesis suggests that higher-income groups support action to protect the environment because they can afford to make financial sacrifices (Gelissen 2007). One study provides evidence that formal education is influential when adopting battery storage (Alipour et al. 2022), presumably because there is a need to seek out information and make an informed decision. Research reports that the older the house owner, the lower the likelihood of retrofit activities (Achtnicht and Madlener 2014). Likewise, a recent study on battery storage found that age was one of the strongest predictors of battery adoption. The older the prospective buyers, the less likely they were to purchase a battery (Poier 2023). An Australian study of households with solar panels concluded that the uptake of batteries is more likely by households with younger occupants (Best et al. 2021). A European study on electric vehicles found that in Nordic Regions, younger, better-educated men, higher-income women and young (affluent) retirees were more likely to adopt electric vehicles (Sovacool, Kester, Noel and de Rubens 2018). Based on the aforementioned studies, the following hypotheses are formulated:

- H6a. Higher income is positively associated with the acceptance of battery storage.
- H6b. A higher educational level is positively associated with the acceptance of battery storage.
- H6c. Older age is negatively associated with the acceptance of battery storage.

## Method

### Data collection, sampling procedure and measurement

Ethical approval for the study was obtained from an Australian university. Data were collected through a web-based survey, and participants were recruited via a professional market research company, Qualtrics. The survey was conducted in 2022. Purposive sampling was used. The target group was homeowners over the age of 18 living in Queensland, Australia, who have the capacity to install battery storage. The state of Queensland was chosen since the state has abundant solar energy resources, and government policy has resulted in high adoption rates of solar PV (Lan et al. 2020; Sommerfeld et al. 2017). Exclusion criteria consisted of people under 18 who were not responsible for paying the electricity bill and renters since home ownership is an important driver of renewable energy adoption (Klingler 2017; Sunter, Castellanos and Kammen 2019).

The dependent variable—acceptance of battery storage—was measured using a scale proposed by Fett et al. (2021). A 7-point Likert scale (ordinal data) was used, where 1 represented ‘strongly disagree’ and 7 represented ‘strongly agree’. The measure for environmental self-identity was taken from research by Barbarossa and De Pelsmacker (2016) and Whitmarsh and O’Neill (2010), which also used a 7-point Likert scale. The survey also included questions on barriers to battery storage adoption, which were informed by government reports and a study on barriers against energy retrofit measures (Achtnicht and Madlener 2014). For this question, a 5-point scale was used, where 1 represented ‘clearly describes my concerns’ and 7 represented ‘does not describe my concerns’. There were also questions on solar PV adoption and load-shifting behaviours (Wittenberg and Matthies 2016). A pilot study was conducted prior to the main survey to check the validity of the measurement scales. In the section on demographics, respondents were asked to answer closed questions (nominal/categorical data). The survey scales and source of the measures are shown in the supplementary data.

Two ‘what-if’ scenario questions were used to measure prosumer scenarios. For the first scenario, respondents were asked to indicate the likelihood of installing batteries in response to a generous FiT used to reward people for sharing and trading electricity and supporting the grid. A 7-point scale (1 = extremely unlikely and 7 = extremely likely) was used. For the second scenario, respondents were asked to rank three purchase options. The items captured attributes such as the price of a battery^1^ (Origin Energy, (n.d.)), the option to export electricity to the grid or use it for self-consumption only, and the option to either fully own or lease the battery, which a third party maintains. For this scenario, a rank-order response category was used. The respondents were asked to select their most preferred to their least preferred option or none.

Quality checks were conducted, and any cases that included unreliable responses or that were performed too quickly were deleted. A total of 609 usable responses were obtained, which included 52 households that had installed battery storage. A final sample size of 557 was used for the regression analysis since the purchase of a system could raise the issue of causality and lead to flawed interpretations. Nemes et al. (2009) show that for studies using regression modelling, larger sample sizes, preferably 500, will increase the accuracy of the estimates. We performed a power analysis (Faul et al. 2007), which indicated that the calculated required sample size for a *t*-test to detect a medium effect size with 80% power and a significance level of 0.05 (two-tailed) is approximately 64 for each group. Given that our sample size far exceeds the required sample size, it has more than adequate power to detect a medium effect size under the given conditions, and this study remains robust against type II errors, even in the context of a two-tailed test. Based on the studies above, a sample size of 557 is reasonable.

### Methods and data analysis

The data obtained from the survey were analysed using STATA 17 and IBM SPSS version 27. A rigorous two-step econometric framework was used. In the initial phase, exploratory factor analysis was used to uncover patterns in the survey responses and transmute raw survey data into latent, unobservable constructs (Field 2013). The insights gained from the factor analysis served as the foundation for an ordered logistic regression test, which were used as the factors that may affect the market acceptance. Considering the ordinal nature of the response (likelihood of 1 to 7) to the query, ‘To what extent do you accept battery storage systems?’, ordered logistic regression was ideally suited to predicting categorical outcomes. A major assumption of ordinal logistic regression is the assumption of proportional odds: the effect of an independent variable is constant for each increase in the level of the response (Greene 2012). The observed response was modelled by conceptualising a latent variable, ${y}_{i}^{*}$, that exhibits a linear dependency on the independent variable $x$:

1 $${y}^{*}={x}_{i}^{\prime}\beta + \mu$$

where ${y}_{i}^{*}$ is an unobserved dependent variable $, {x}_{i}{\prime}$ s the vector of independent variables, $u$ is the error term, assumed to follow a standard logistic distribution, and β is the vector of regression coefficients. The observed ${y}_{i}$ is based on ${y}_{i}^{*}$ according to the rule:

2 $$y_i=\;\left\{\begin{array}{ccc}0&if&y_i^\ast\leq\gamma_1\\1&if&\gamma_1<y_i^\ast\leq\gamma_2\\2&if&\gamma_2<y_i^\ast\leq\gamma_3\\\vdots&\vdots&\vdots\\M&if&\gamma_M<y_i^\ast\end{array}\right.$$

The dependent variable ${y}_{i}$ in the model refers to the extent of battery storage acceptance while ${x}_{i}$ represents a set of independent variables, including gender, age, income, education, norms, environmental self-identity, perceived barriers and other variables derived from the factor analysis.

Reliability and validity tests were undertaken (using Smart PLS, version 4) to test the quality of the survey measures, bolster the results of the factor analysis and ensure empirical robustness. In order to answer the final research question and compare the prosumer with non-prosumers, the research employed the Pearson’s chi-square test and the independent sample *t*-test. The Pearson chi-square test is a statistical test used to determine if there is a relationship between two categorical variables (Field 2013), such as being a prosumer or a non-prosumer and falling into a particular demographic category. The independent sample *t*-test is a statistical test that compares the mean values of two independent samples (Field 2013), and it was used to compare the attitudes of the prosumer with those of non-prosumers in relation to the acceptance of battery storage and perceived barriers to installing storage.

## Results

### Description of the sample

Descriptive statistics are presented as supplementary data (Appendix A, Table A1). More women (60.9%) than men participated in the survey. Respondents came from different age groups, with the majority falling into the older age brackets, reflecting the pattern of home ownership in Australia: 36–45 years (13.5%), 46–55 years (13.1%), 56–65 years (18.7%) and 66–75 years (26.4%). The respondents’ level of educational attainment varied, with 27.3% having a bachelor’s degree. Close to half of the sample (47.6%) were employed or self-employed. Household income was dispersed across all income categories, with the majority falling into two income brackets: AUD$30,000 to $64,999 (28.9%) and AUD$65,000 to $99,999 (21.8%). The type of household that was most frequently reported was a two-person household.

**Table 1:** Consumers’ preferred options concerning battery purchase

	1	2	3	0
Option	% (*n*)	% (*n*)	% (*n*)	% (*n*)
Owned outright: AUD$10,000 (after government subsidy), option to export solar and get credit	30.8 (171)	21.4 (119)	20.2 (112)	23.2 (129)
Owned outright: AUD$8000, limited option to export since the system is for self-consumption	17.7 (98)	40.2 (223)	18.6 (103)	23.1 (128)
Leased: discounted purchase price of AUD$5000, third party manages battery and exports	24 (133)	13.5 (75)	35.7 (198)	23.4 (130)

1 = ranked as first preference to 3 = ranked as last preference; 0 = none of the above

### Descriptive statistics on battery storage

Concerning low-carbon technologies, approximately half of the sample had rooftop solar (50.4%), and a small percentage of homeowners, 8.5% (*n* = 52), had battery storage installed at home. Non-adopters of battery storage were provided with a list of reasons to explain why they would consider installing battery storage systems, and multiple answers were allowed. Respondents reported that lack of money, inadequate government subsidies and the long payback period ‘moderately describe’ their concerns. Safety, technological change and the risk of not picking the best storage option were said to ‘slightly describe’ people’s concerns. A feeling that battery storage is not necessary was found to ‘slightly describe’ people’s beliefs. Receiving the premium FiT received the lowest mean score. Further descriptive statistics on barriers to adopting storage are presented as supplementary data (Appendix A, Table A2).

**Table 2:** Likelihood of installing battery storage under a prosumer scenario

Scenario			
Batteries can be charged by solar panels on the roof. Since they store electricity, they make solar energy less dependent on the weather and could meet a home’s daily energy needs. Surplus electricity could be sent to the national grid, earning money for homeowners. A feed-in tariff (FiT) is a credit people can receive when excess energy is returned to the grid. Batteries also support the grid, making it more secure and reliable. A new incentive policy, a generous FiT rate, has been introduced to promote battery storage and reward people for sharing and trading electricity. How likely would you be to install battery storage under this scenario?			
(*n* = 557)	*n*	%	Mean: 4.7540 Standard deviation: 1.7286
Extremely unlikely (= 1)	50	9	
Moderately unlikely	28	5	
Slightly likely	13	2.3	
Neither likely nor unlikely	131	23.6	
Slightly likely	109	19.5	
Moderately likely	148	26.6	
Extremely likely (= 7)	78	14.0	

A 7-point scale was used, where extremely unlikely = 1 and extremely likely = 7

### Prosumer scenario: ownership or leasing options

One of the research objectives was to harness insight into consumer preferences for battery storage attributes, considering the price, ownership or leasing options, and the option to export surplus electricity back to the grid. Table 1 displays the proportion of ranked priorities for the three scenarios.

Respondents’ preferences for the three battery scenarios varied greatly. Almost one-third of the sample (30.8%) selected outright ownership, the relatively high price and the option to export solar as their first preference. Leasing was a popular option, with 133 respondents (24%) ranking it as their first preference. Only 17.7% of the sample considered the moderately priced, self-consumption system as their first preference.

Regarding the top two preferences, outright ownership at the lower price was perceived as important, with 321 respondents (57.9%) ranking this in their top two preferences. Outright ownership at the higher price with export was also ranked in the top two by more than half of the sample (52.2%). Leasing was ranked lower, with 208 respondents (37.5%) ranking it among their top two preferences. Alternatively, survey participants were given the option not to rank the options, and approximately 23% of respondents indicated ‘none of the above’.

### Prosumer scenario: trading and sharing electricity under a feed-in tariff regime

One research objective was to evaluate attitudes towards a prosumer scenario. Respondents were asked to indicate the likelihood of installing batteries if a generous FiT rate was introduced to reward people for sharing and trading electricity (see Table 2). The mean value was 4.75 (slightly likely), indicating a willingness to act as a prosumer. Approximately one out of four respondents (26.6%) said they were moderately likely to install batteries under this scenario, and a small percentage (14%) were extremely likely to act as prosumers.

One research objective was to compare prosumers with non-prosumers. The results of the *t*-test (presented as supplementary data, Appendix A, Table A4) illustrated that prosumer households show higher acceptance of battery storage and exhibit fewer concerns about batteries than non-prosumers. The results of the chi-square analyses, including the effect size, are presented as supplementary data (Appendix A, Table A5). There were significant relationships between prosumerism and the installation of rooftop solar: *χ*^2^ (1, 257] = 10.504, *p* < 0.001. Chi-square tests showed significant differences between prosumers and non-prosumers in respect to demographics, such age: χ^2^ [2, 557] = 7.222, *p* = 0.027; education: χ^2^ [2, 557] = 9.036, *p* = 0.011, income: χ^2^ [3, 520] = 16.343, *p* = 0.001, and household size: χ^2^ [3, 557] = 7.819, *p* = 0.050.

### Construct reliability and validity

Table 3 displays the findings for internal consistency and convergent validity. Data are presented for the multiple-item constructs only: those that measure the dependent variable and several of the independent variables. The constructs have a high level of reliability and internal consistency, with Cronbach’s alpha values above the recommended value of 0.7 and a rho_A value of higher than 0.7 and less than 1. The composite reliability values exceeded the threshold value of 0.7 (Bagozzi and Yi, 1988). The convergent validity measure comprises the average variance extracted, which surpassed the threshold value of 0.5 (Bagozzi and Yi, 1988). The table also summarises the result for the discriminant validity test—the hetero-trait–mono-trait criterion (presented as supplementary data, Appendix A, Table A6). The analysis showed that no value was close to 1, and all values were below the recommended threshold of 0.85 or 0.90 (Benitez et al. 2020).

**Table 3:** Construct reliability and validity for multiple-item scales

Variable, scale items	Cronbach’s alpha	rho_A	Composite reliability	Average variance extracted	Hetero-trait–mono-trait ratio < 0.085?
Acceptance of battery storage I can imagine using a photovoltaic (PV) battery storage system I would like to use a PV battery storage system Investing in a PV battery storage system has more advantages than disadvantages I consider PV battery storage systems to be sensible and sustainable I can imagine investing in a PV battery storage system	0.944	0.952	0.957	0.817	Yes
Moral emotions I would feel guilty if I did not save electricity on a daily basis My conscience would bother me if I did not save electricity on a daily basis	0.873	0.919	0.939	0.885	Yes
Energy-positive attitude Saving electricity is useless Saving electricity is good I am more willing to buy an appliance with an efficient energy class	0.744	0.748	0.854	0.66	Yes
Environmental self-identity I would be embarrassed not to be seen as having an environmentally friendly lifestyle I think of myself as someone who is concerned with environmental issues I see myself as environmentally friendly	0.863	0.863	0.917	0.788	Yes

### Factor analysis

Exploratory factor analysis was used since the aim was to explore the data rather than test a specific hypothesis. Principal component analysis was the chosen method, as indicated in Table 4. The data showing the significant item loadings can be found in the supplementary data (Appendix A, Table A7). Descriptive statistics on all the factors used in the regression model, such as the mean and standard deviation values, can also be found in the supplementary data (Appendix A, Table A8). The results of the factor analysis are shown in Table 4.

**Table 4:** Results of the factor analysis

Factor	Eigenvalue	Difference	Proportion	Cumulative
Factor 1	5.1338	2.7984	0.2445	0.2445
Factor 2	2.3354	0.3339	0.1112	0.3557
Factor 3	2.0015	0.7129	0.0953	0.4510
Factor 4	1.2886	0.1572	0.0614	0.5123
Factor 5	1.1314	0.1388	0.0539	0.5662
Factor 6	0.9926	0.0897	0.0473	0.6135
Factor 7	0.9029	0.0900	0.0430	0.6565
Number of observations	557			
Method	Principal component factors			
Retained factors	5			
Rotation	Unrotated			
Number of parameters	95			

We conducted a factor analysis that included several constructs from the survey, including subjective norms and moral emotions, positive attitudes towards energy-saving, self-identity, energy-related motives and perceived barriers. As shown in Table 4, the Eigenvalues for five factors (factors 1–5) were greater than 1; therefore, these factors were retained. Correlation and sampling adequacy tests, notably the Kaiser–Meyer–Olkin measure of sampling adequacy (KMO), were performed. After factor analysis is performed, Kaiser (1974) recommends assessing how good the solution is in terms of simplicity. The general rule is to accept values greater than .5 and values below this threshold are unacceptable. The results were satisfactory and are available as supplementary data (Appendix A, Table A9).

The first component heavily weights variables related to affective elements such as self-identity and moral emotions; the second weights variables related to energy-positive attitudes, including subjective norms; the third weights variables related to energy-related motives (i.e. related to independence and interest in using smart meters and monitoring consumption), the fourth weights variables about the perception of barriers (i.e. related to safety, the technology and cost) and the fifth weights variables related to thermal comfort.

### Logistic regression

The results of the logistic regression concerning the probability of accepting battery storage are presented in Table 5. As shown in the table, the factors that were statistically significant and positive predictors of battery storage acceptance were affective elements such as self-identity and moral emotions (factor 1), positive attitudes towards energy saving and subjective norms (factor 2), energy-related motives (factor 3) and education levels. Age and perceptions of barriers were statistically significant. The coefficient for age was negative and showed that the probability of accepting storage decreases in older generations. The coefficient for the perception of barriers was negative and showed that as the perception of barriers increases, the likelihood of accepting battery storage decreases. Gender, income and thermal comfort were not statistically significant predictors. The likelihood ratio, chi-square statistics and the pseudo-*R*^2^ value are reported at the bottom of the table. The value of pseudo-*R*^2^ was not large, and the reasons for the low value are as follows: first, fewer predictors are used in the model. Second, unlike the *R*^2^ in linear regression, which ranges between 0 and 1, there is no absolute ‘good’ or ‘bad’ value for it in the logistic model because it is more about comparing models. A higher pseudo-*R*^2^ usually indicates a better fit, but even a low value does not necessarily mean the model is unfit. Third, McFadden’s pseudo-*R*^2^ tends to yield much lower values than expected (Field 2013). However, while pseudo-*R*^2^ can be informative, over-relying on it in logistic regression might be misleading. It is essential to look at the significance of individual predictors and consider the practical implications of the model.

**Table 5:** Results of the regression analysis: antecedents of battery storage acceptance

Variables	Coefficient	Standard error	*z*	*p* > *z*
Factor 1: Identity, moral emotions	0.1965	0.0797	2.46	0.014**
Factor 2: Energy-positive attitudes and norms	0.3139	0.0789	3.98	0.000***
Factor 3: Energy-related motives	0.9027	0.0872	10.36	0.000***
Factor 4: Perception of barriers	–0.3314	0.0794	–4.17	0.000***
Factor 5: Thermal comfort priority	0.0786	0.0807	0.97	0.330
Gender	–0.2505	0.1637	–1.53	0.126
Age	–0.1369	0.0513	–2.67	0.008***
Education	0.1252	0.0578	2.16	0.030**
Income	0.0227	0.0406	0.56	0.575
Number of observations	557.00			
Likelihood ratio chi-square (9)	172.42			
Probability > chi-square	0.00			
Log-likelihood	–883.7938			
Pseudo-*R*^2^	0.0889			

*** indicates significance at the 1% level (*p* < 0.01), ** indicates significance at the 5% level (*p* < 0.05)

## Discussion

### Theoretical implications

Small-scale energy technologies, such as solar and battery storage, are important to the energy sector’s decarbonisation (International Energy Agency 2023) and hold promise in mitigating climate change, particularly considering the rapid cost reductions and performance improvements (Dhakal et al. 2022). Since homeowners play a critical role in the uptake of low-carbon technologies, a deep understanding of the factors that drive acceptance is needed. The primary objective of this study was to examine the social–psychological, behavioural and demographic determinants of battery storage acceptance. The main conclusion of this study is that viewing energy storage purely as an economic decision influenced by price and government subsidies will miss opportunities to target broader segments based on psychological and demographic factors.

It was hypothesised (H1) that perceptions of barriers negatively influence acceptance of battery storage, which was confirmed. Regression analysis showed that concerns about battery technology and costs were negatively associated with acceptance. The analysis showed that subjective norms, moral emotions and an environmental self-identity were positively related to acceptance, confirming H2, H3 and H4. In relation to motives, regression analysis showed that the desire for independence, an interest in using smart meters and monitoring consumption to avail of cheaper tariffs were positively related to acceptance, confirming H5a, H5b and H5c. Regarding demographics, higher levels of education were positively related to acceptance of battery storage, and older age was negatively associated with acceptance, confirming hypotheses H6b and H6c. By scrutinising relatively underexplored concepts in the battery storage literature, our work is a pertinent addition to the ongoing scholarly debates. Reinforcing the significance of our research, Poier (2023 p. 2) contends, ‘although the adoption of photovoltaic (PV) systems has been extensively analysed, the realm of electricity storage adoption remains comparatively under-researched’.

The finding on the significance of self-identity for acceptance is aligned with the literature. Prior research shows a link between self-identity and pro-environmental behaviours (such as energy use, waste, transport and shopping choices) (Whitmarsh and O’Neill 2010). Other studies reveal a link between self-identity and climate action (Bouman et al. 2021) and between self-identity and sustainable consumption (such as the purchase of eco-friendly paper products) (Barbarossa and De Pelsmacker 2016). Prior research shows that although government subsidies reduce consumers’ concerns about paying a premium price for new energy technologies, willingness to pay is strongly affected by environmental concern (Shao and Ünal 2019). This study confirms the link between subjective norms and behaviour outlined in the seminal theory of planned behaviour (Ajzen 1991). It is an important finding since the literature presents conflicting results. The finding that moral emotions influence behaviour is consistent with a recent meta-analysis on the role of guilt in motivating pro-environmental behaviour (Hurst and Sintov 2022). It is found that having information about the impact of fossil fuels on the environment tends to induce guilt and a moral obligation to invest in new energy technologies, in line with the norm activation model (He and Zhan 2018). Similar to other studies, the desire for independence (Agnew and Dargusch 2017) and less reliance on electricity suppliers are predictors of battery storage acceptance (Bondio et al. 2018). Prior studies have found that technical interest is a motive for battery acceptance (Hackbarth and Löbbe 2020), which is somewhat aligned with our finding that an interest in smart meters and monitoring consumption predicts acceptance.

Concerning demographics, we hypothesised that battery storage acceptance would be influenced by education and age (H6b and H6c), which was confirmed. It stands to reason that education is influential since decision-making related to energy storage is complex and involves considering payback periods, electricity-saving potential, access to funding and the safety and durability of the battery. Prior research notes the importance of education (Alipour et al. 2022) and age (Poier 2023). The significance of older age in negatively influencing the acceptance of batteries warrants an explanation. The literature on ageing tends to depict older adults as problematic and passive recipients of technology (Peine et al. 2014). Despite such stereotypes, ageing is found to be a negative factor in relation to the adoption of smart home technology due to the existence of the digital divide (Li et al. 2022). The decreased income as people approach retirement could also make battery purchases less attractive. The risk of illness or the need to move out of the house could also mean that older people might avoid making a long-term investment. Older adults are the fastest-growing segment of the global population, and studies link population ageing with energy consumption (Yu, Wei, Gomi and Matsuoka 2018); thus, the lack of battery storage acceptance among older age groups should concern policymakers. Older consumers might find it more difficult than younger consumers to accept the high price of battery storage and possible risks, even if they have an environmental self-identity. Surprisingly, regression analysis showed that acceptance of battery storage is not sensitive to income, which contradicts previous literature on renewable energy adoption (Bondio et al. 2018; Brown 2022). One explanation for this finding is the complexity of the substitution and income effects on different households. For example, on the one hand, high-income households may not care about an expensive electricity bill, so batteries are not an attractive investment. On the other hand, low-income households may care about the bill but cannot afford the capital cost of battery installation.

We hypothesised that thermal comfort demand (H5d), a behavioural variable, would influence the acceptability of storage (H5d), which was not confirmed. However, this factor may be too distant or isolated from the decision to invest in battery storage, and a distinction can be drawn between home-based, daily practices and investment.

The results show that perceived barriers are negatively related to acceptance, and this is aligned with prior research highlighting high costs, long payback periods (van Groenou et al. 2018), as well as safety concerns (Agnew and Dargusch 2017; Kalkbrenner 2019). These findings are important since they reveal strategies that could improve acceptance.

A second objective of this study was to evaluate consumers’ attitudes towards battery leasing and ‘prosumer’ models. According to Kalkbrenner (2019, p.1,355), ‘…little is known about consumer preferences and appropriate business models for storage systems’. Thus, this investigation holds paramount importance, especially since research on Australian battery storage consumers is limited, and the commercial models surrounding these paradigms remain ambiguous. The study revealed that one-quarter of the sample was interested in leasing batteries and actively participating in energy markets. Prosumer households showed higher acceptance of battery storage and exhibited fewer concerns about batteries than the non-prosumers. In addition, the results showed significant relationships between prosumerism and the installation of rooftop solar, age, income, education and household size. This result is not surprising since studies of rooftop solar adopters also highlight demographic variables, and the early adopter is typically affluent and middle-class (Nelson et al. 2011).

### Implications for policy and practice

Policymakers and practitioners in other countries should fully consider the role of non-economic factors in the promotion of residential battery storage. With the decline in government subsidies for installing rooftop solar and battery storage, interventions that appeal to affective and social influences are likely to be effective. One method of appealing to subjective norms is to make the invisible behaviour (e.g. installation of batteries inside the home) more visible. This could be achieved by encouraging buyers to display a sticker indicating their support of low-emissions technology (e.g. ‘I am a supporter of battery storage technology’) or to use customised number plates (e.g. showing symbols and the colour green). This study unveiled the often-overlooked affective influences on battery storage acceptance. Thus, it may be worthwhile for businesses to craft narratives and taglines that are aligned with moral emotions (i.e. ‘I feel better about using electricity with storage’) and an environmental self-identity (i.e. ‘I support storage—not fossil-fuel lifestyles that damage the environment’). The regression analysis revealed that acceptance is related to positive attitudes towards energy saving, along with a belief that ‘people who are important to me think that I should save electricity’. Therefore, marketing approaches that encourage interpersonal communication and build social proof (such as offering discounts for referrals and using customer testimonials on websites and social channels) would also be helpful. Strengthening the effect of identity and norms in the marketplace may act to bolster confidence and change attitudes about the costs and drawbacks of adopting battery storage. Since the desire for independence predicts acceptance, businesses could devise appealing storylines that appeal to storage-related motives in marketing campaigns.

The significance of older age in negatively influencing the acceptance of batteries holds lessons for public policymakers. The findings on age provide a new perspective and opportunity for policymakers to differentiate marketing communications based on age and target older adults in order promote a just transition. Older adults may also need reassurance that battery storage systems are safe and that they are making the right choice. Being mindful that subsidies and FiTs can deepen social inequities and that groups experiencing fuel poverty are underrepresented in policymaking (Best et al. 2021; Sovacool, Martiskainen, Hook and Baker 2019), incentives to improve the affordability of batteries should be carefully targeted. Hence, capital subsidies targeted at older age groups, particularly those from low socio-economic groups who cannot afford to install battery storage, could be promoted on energy justice grounds.

The regression analysis shows that as the perception of barriers increases, the likelihood of accepting battery storage decreases. The practical implications of this research for business actors are that the appeal of battery storage may be limited without stronger marketing and after-sales service. To assuage concerns about the safety of battery storage technology, we recommend investment in after-sales service and the development of an insurance scheme. Retailers could boost confidence by providing maintenance service and offering warranties in the unlikely event that the battery storage system could fail or pose a safety hazard. Consumers are concerned about not choosing the best storage system and perceive that the payback period is too long. To reassure consumers, the benefits of lithium-ion technologies, such as decreasing prices, higher energy efficiencies and longer lifetimes (Figgener et al. 2020), should be communicated.

One survey question examined consumers’ attitudes towards business models that incentivise people to act as prosumers. The results suggest that offering energy consumers more choice may help bolster the growth of the residential battery storage market. For instance, outright ownership of a solar system with solar export could appeal to consumers who wish to profit from their systems. The leasing of a discounted system (with solar exports managed by a third party) could help some consumers overcome the cost barrier to purchase (with the added benefit of assisting the grid in managing peak demand). Utilities could cater for the interest in prosumerism by embracing new business models. Chi-square analysis revealed significant differences between prosumers and non-prosumers with respect to demographics. Targeting prosumer households on the basis of age (36 to 56 years), education (post primary), income (greater than $65,000), household size (greater than 3 persons) and the prior installation of solar systems may be worthwhile, with the caveat that further research based on robust segmentation techniques is needed. It is worth mentioning that many factors are stalling the development of local energy trading markets, such as transaction costs (Schwidtal et al. 2023) and regulatory issues arising from the prohibition of direct energy exchanges between prosumers in many countries (Sousa et al. 2019).

### Limitations, future research and conclusions

Several limitations warrant attention. One is the potential for socially desirable responses, even though anonymity was guaranteed. Another potential limitation is the selection of participants via a paid consumer panel, which carries the risk of selection bias. The survey focused on general feelings of acceptance towards batteries that may not correspond with actual behaviour. Further research should investigate alternative households, such as renters, apartment dwellers and low-income households, who could benefit from landlord–tenant electricity agreements, leasing models and community or shared electricity storage within a neighbourhood. Given the significance of age, further research, drawing on the concept of generation/cohort effects (Li 2015), is needed to compare the perceptions of younger generations with older generations and explore the factors that influence battery storage acceptance. Notwithstanding these limitations, this study makes an important empirical contribution to the energy literature since the antecedents of battery storage are not well understood. Battery storage by private consumers helps resolve the intermittent nature of renewable energy, can relieve pressure on power grids and contributes to climate change mitigation efforts. Hence, the topic is worthy of empirical research and contributes to discussions and debates in the literature.

## Supplementary Information

Below is the link to the electronic supplementary material.

Supplementary file1 (DOCX 51 KB)

## Data Availability

Available on request.

## Footnotes

1 As of late 2022, a Tesla Powerwall 2 can be installed for around AUD$14,995, and prices are forecasted to fall considerably in the future. The pricing options in the survey were influenced by the information displayed on a major electricity retailer’s website.
