# Supplementary material for: It starts at home: non-economic factors influencing consumer acceptance of battery storage in Australia
Source: Environ Sci Pollut Res Int. 2024 Feb 27;31(46):57129–45. doi: 10.1007/s11356-024-32614-5 (PMC11481664; doi:10.1007/s11356-024-32614-5)
Supplement: Supplementary file 1 — Supplementary file1 (DOCX 51 KB) [file 11356_2024_32614_MOESM1_ESM.docx]

**Online Appendix A**

Table of Contents Page

**Table A1**: Sample statistics 2

**Table A2**: General concerns about battery storage 3

**Table A3**: Prosumers and non-prosumers: descriptive statistics 4

**Table A4**: Prosumers and non-prosumers: independent sample t-tests 5

**Table A5**: Results of the Chi-Square analysis: characteristics of

prosumer households 6

**Table A6:** Discriminant validity (the HTMT test) 7

**Table A7**: Factor loadings 8

**Table A8**: Factors used in the regression model, descriptive statistics 9

**Table A9:** Correlation and sampling adequacy test 10

**Table A1** Sample Statistics (n=609)

|  | **Demographics** | No. | Percent |
| --- | --- | --- | --- |
| Gender | Male | 238 | 39.1 |
|  | Female | 371 | 60.9 |
| Age | 18-25 | 30 | 4.9 |
|  | 26-35 | 71 | 11.7 |
|  | 36-45 | 82 | 13.5 |
|  | 46-55 | 80 | 13.1 |
|  | 56-65 | 114 | 18.7 |
|  | 66-75 | 161 | 26.4 |
|  | 76+ years | 71 | 11.7 |
| Employment | A student | 4 | .7 |
|  | Employed | 252 | 41.4 |
|  | Self-employed | 38 | 6.2 |
|  | Unemployed | 29 | 4.8 |
|  | Looking after home or family | 49 | 8.0 |
|  | Retired | 237 | 38.9 |
| Education | Primary school, or no formal qualification | 34 | 5.6 |
|  | High school certificate | 150 | 24.6 |
|  | Trade or vocational qualification | 105 | 17.2 |
|  | Diploma or advanced diploma | 99 | 16.3 |
|  | Undergraduate degree (Bachelor's) | 166 | 27.3 |
|  | Postgraduate degree (Masters, PhD) | 55 | 9.0 |
| Income | Less than $30,000 | 83 | 13.6 |
|  | $30,000 to $64,999 | 176 | 28.9 |
|  | $65,000 to $99,999 | 133 | 21.8 |
|  | $100,000 to $149,000 | 94 | 15.4 |
|  | $150,000 to $199,000 | 50 | 8.2 |
|  | $200,000 to $249,000 | 22 | 3.6 |
|  | $250,000 to $299,999 | 7 | 1.1 |
|  | More than $300,000 | 5 | .8 |
|  | Do not know/prefer not to say | 39 | 6.4 |
| Household size | 1 person | 103 | 16.9 |
|  | 2 persons | 288 | 47.3 |
|  | 3 persons | 112 | 18.4 |
|  | 4 persons | 70 | 11.5 |
|  | 5 persons or more | 36 | 5.9 |

**Table A2** General concerns about battery storage

| Statement | Mean | Median | Std Dev. |
| --- | --- | --- | --- |
| **Barriers /concerns about batteries**  (n=557) |  |  |  |
| I/we are lacking the financial resources. | 3.00 | 3.00 | 1.519 |
| Adequate government subsidies are not available. | 2.96 | 3.00 | 1.405 |
| I don't think the investment will pay off / payback period is too long. | 2.78 | 3.00 | 1.372 |
| I am apprehensive of technological change and the risk of not picking the best storage option. | 2.41 | 2.00 | 1.246 |
| I am concerned about safety. | 2.19 | 2.00 | 1.258 |
| Battery storage is not necessary. | 2.16 | 2.00 | 1.180 |
| My/our house is already energy optimized. | 2.02 | 2.00 | 1.159 |
| I am not sure how much longer I will stay in this house. | 1.95 | 1.00 | 1.269 |
| I am getting a premium feed-in tariff of 44c for my electricity and have no incentive to store it. | 1.81 | 1.00 | 1.230 |

*Note:* a 5-point scale used, where 1= does not describe my concerns to 5=clearly describes my concerns.

**Table A3** Prosumers and non-prosumers: descriptive statistics

|  | **Prosumer**  **(n= 226)** | | **Non-Prosumer**  **(n=331)** | |
| --- | --- | --- | --- | --- |
| **Statements about concerns/ barriers to battery storage adoption** | **Mean** | **Std D** | **Mean** | **Std D** |
| I am not sure how much longer I will stay in this house | 1.77 | 1.77 | 2.08 | 2.08 |
| I don't think the investment will pay off / payback period is too long. | 2.45 | 2.45 | 3.01 | 3.01 |
| I/we are lacking the financial resources. | 2.88 | 2.88 | 3.09 | 3.09 |
| My/our house is already energy optimized. | 1.85 | 1.85 | 2.14 | 2.14 |
| I am concerned about safety | 2.02 | 2.02 | 2.31 | 2.31 |
| I am apprehensive of technological change and the risk of not picking the best storage option. | 2.24 | 2.24 | 2.53 | 2.53 |
| I am getting a premium feed-in tariff of 44c for my electricity and have no incentive to store it | 1.73 | 1.73 | 1.86 | 1.86 |
| Adequate government subsidies are not available. | 3.06 | 3.06 | 2.89 | 2.89 |
| Battery storage is not necessary. | 1.84 | 1.84 | 2.38 | 2.38 |
| **Acceptance of battery storage** |  |  |  |  |
| I can imagine using a PV battery storage system. | 5.49 | 5.49 | 3.92 | 3.92 |
| I would like to use a PV battery storage system. | 5.69 | 5.69 | 4.01 | 4.01 |
| Investing in a PV battery storage system has more advantages than disadvantages | 5.37 | 5.37 | 3.95 | 3.95 |
| I consider PV battery storage systems to be sensible and sustainable. | 5.52 | 5.52 | 4.15 | 4.15 |
| I can imagine investing in a PV battery storage system. | 5.31 | 5.31 | 3.76 | 3.76 |

*Note^1^*: the scale for concerns/barriers to battery storage adoption ranges from 1=does not describe my concerns to 5= clearly describes my concerns. The scale for battery storage acceptance ranges from 1=strongly agree to 7=strongly disagree.

*Note^2^:* the prosumer group was coded 1=likely to engage in electricity sharing and trading under a feed-in tariff regime; the non-prosumer group was coded 2=unlikely to engage in electricity sharing and trading (categorical variable).

**Table A4** Prosumers and non-prosumers: independent samples t-test

|  |  | Equality of Variances | | t-test for Equality of Means | | | | | Cohen’s D |
| --- | --- | --- | --- | --- | --- | --- | --- | --- | --- |
|  |  | F | Sig. | t | df | Sig. (2-tailed) | Mean Diff. | Std. Error Diff |  |
| I am not sure how much longer I will stay in this house | Equal variances assumed | 11.696 | 0.001 | 2.877 | 555 | 0.004 | 0.313 | 0.109 | .248 |
|  | Equal variances not assumed |  |  | 2.964 | 528.290 | 0.003 | 0.313 | 0.106 |  |
| I don't think the investment will pay off / payback period is too long. | Equal variances assumed | 3.363 | 0.067 | 4.870 | 555 | 0.000 | 0.565 | 0.116 | .420 |
|  | Equal variances not assumed |  |  | 4.982 | 518.999 | 0.000 | 0.565 | 0.113 |  |
| I/we are lacking the financial resources. | Equal variances assumed | 0.029 | 0.864 | 1.616 | 555 | 0.107 | 0.212 | 0.131 | .139 |
|  | Equal variances not assumed |  |  | 1.617 | 485.105 | 0.106 | 0.212 | 0.131 |  |
| My/our house is already energy optimized. | Equal variances assumed | 8.114 | 0.005 | 2.883 | 555 | 0.004 | 0.286 | 0.099 | .249 |
|  | Equal variances not assumed |  |  | 2.965 | 526.169 | 0.003 | 0.286 | 0.097 |  |
| I am concerned about safety | Equal variances assumed | 5.453 | 0.020 | 2.718 | 555 | 0.007 | 0.293 | 0.108 | .235 |
|  | Equal variances not assumed |  |  | 2.749 | 501.581 | 0.006 | 0.293 | 0.107 |  |
| Apprehensive of technological change and risk of not picking the best storage option. | Equal variances assumed | 1.214 | 0.271 | 2.669 | 555 | 0.008 | 0.285 | 0.107 | .230 |
|  | Equal variances not assumed |  |  | 2.688 | 495.587 | 0.007 | 0.285 | 0.106 |  |
| Getting a premium feed-in tariff of 44c for electricity and have no incentive to store it | Equal variances assumed | 2.724 | 0.099 | 1.262 | 555 | 0.207 | 0.134 | 0.106 | .109 |
|  | Equal variances not assumed |  |  | 1.279 | 504.769 | 0.201 | 0.134 | 0.105 |  |
| Adequate government subsidies are not available. | Equal variances assumed | 0.529 | 0.468 | -1.410 | 555 | 0.159 | -0.171 | 0.121 | -.122 |
|  | Equal variances not assumed |  |  | -1.418 | 493.083 | 0.157 | -0.171 | 0.120 |  |
| Battery storage is not necessary. | Equal variances assumed | 6.310 | 0.012 | 5.453 | 555 | 0.000 | 0.541 | 0.099 | .471 |
|  | Equal variances not assumed |  |  | 5.594 | 522.921 | 0.000 | 0.541 | 0.097 |  |
| I can imagine using a PV battery storage system. | Equal variances assumed | 10.789 | 0.001 | -12.998 | 555 | 0.000 | -1.573 | 0.121 | -1.122 |
|  | Equal variances not assumed |  |  | -13.748 | 551.841 | 0.000 | -1.573 | 0.114 |  |
| I would like to use a PV battery storage system. | Equal variances assumed | 28.328 | 0.000 | -13.744 | 555 | 0.000 | -1.681 | 0.122 | -1.186 |
|  | Equal variances not assumed |  |  | -14.934 | 551.005 | 0.000 | -1.681 | 0.113 |  |
| Investing in a PV battery storage system has more advantages than disadvantages | Equal variances assumed | 2.887 | 0.090 | -12.168 | 555 | 0.000 | -1.426 | 0.117 | -1.050 |
|  | Equal variances not assumed |  |  | -12.716 | 543.575 | 0.000 | -1.426 | 0.112 |  |
| I consider PV battery storage systems to be sensible and sustainable. | Equal variances assumed | 9.650 | 0.002 | -11.762 | 555 | 0.000 | -1.371 | 0.117 | -1.015 |
|  | Equal variances not assumed |  |  | -12.435 | 551.612 | 0.000 | -1.371 | 0.110 |  |
| I can imagine investing in a PV battery storage system. | Equal variances assumed | 14.505 | 0.000 | -12.824 | 555 | 0.000 | -1.551 | 0.121 | -1.107 |
|  | Equal variances not assumed |  |  | -13.500 | 549.145 | 0.000 | -1.551 | 0.115 |  |

**Table A5** Results of the Chi-Square analysis: association between prosumer and non-prosumer households, solar system and demographics.

| **Variables** | **Results from Pearson’s chi-square test** | **Categorical variables** |
| --- | --- | --- |
| Grouping variable | Prosumerism | The prosumer group was coded 1=likely to engage in electricity sharing and trading under a feed-in tariff regime; the non-prosumer was coded 2=unlikely to engage in electricity sharing and trading. |
| **Solar system adoption and size** |  |  |
| Installation of rooftop solar | χ2 [1, 257] = 10.504, p = .001***, Cramer’s ν = .137. | Solar installation was measured using a binary variable, coded 1=yes and 2=no. |
| Size of solar system | χ2 [1, 257] = 2.737, p =.098*, Cramer’s ν = .103 | Size of solar system was recoded into two categories such as 1=small to medium (9kW or under) and 2= large (10 kW or over). |
| **Demographics** |  |  |
| Age | χ2 [2, 557] = 7.222, p = .027**, Cramer’s ν = .114 | Age was recoded into three categories where 1=young (35 years and under), 2=middle (36-55 years) and 3=senior (56 years and older). |
| Education | χ2 [2, 557] = 9.036, p = .011**, Cramer’s ν = .127 | Education was recoded into three categories where 1=primary or high school, 2=trade, vocational or diploma and 3= degree, undergraduate or postgraduate qualification. |
| Income | χ2 [3, 520] = 16.343, p = .001***, Cramer’s ν = .177). | Income was recoded into four categories where 1=low (<$30,000), 2=medium ($30,000 to $64,999), 3=high ($65,000 to $99,999) and 4=very high (>100,000). |
| Household size | χ2 [3, 557] = 7.819, p =.050*, Cramer’s ν = .118 | Household size was recoded into four categories where 1=single household, 2=dual-person household, 3=3 or 4 persons and 4=more than 4 persons in the household. |

*Note:* Significant at:*p<0.10 (less than 10%), ** p < 0.05 (less than 5%) and *** p < 0.01 (less than 1%).

**Table A6** Discriminant validity (the HTMT test)

|  | Acceptance of battery storage | Energy positive attitudes | Environmental self-identity | Moral norms |
| --- | --- | --- | --- | --- |
| Acceptance of battery storage |  |  |  |  |
| Energy positive attitudes | 0.294 |  |  |  |
| Environmental self-identity | 0.307 | 0.631 |  |  |
| Moral norms | 0.206 | 0.534 | 0.632 |  |

**Table A7** Factor Loadings

| No. | Variable | Factor 1 | Factor 2 | Factor 3 | Factor 4 | Factor 5 | Uniqueness |
| --- | --- | --- | --- | --- | --- | --- | --- |
| Q10_2 | Payback period | -0.1561 | -0.0417 | 0.0035 | **0.6062** | -0.2152 | 0.5601 |
| Q10_5 | Safety concern | 0.1402 | -0.0282 | -0.1395 | **0.7730** | 0.1242 | 0.3471 |
| Q10_6 | Technological change | 0.0706 | 0.0228 | 0.0603 | **0.8149** | 0.0428 | 0.3249 |
| Q10_8 | Subsidies | -0.2135 | 0.0805 | 0.2191 | **0.5847** | -0.1827 | 0.5247 |
| Q13 1 | Unconscious use | -0.3639 | 0.2504 | 0.0972 | -0.1650 | 0.4184 | 0.5932 |
| Q14 2 | Turn off lights | -0.3195 | 0.5508 | 0.0409 | -0.0419 | -0.3439 | 0.4728 |
| Q14_3 | Reduce air con | -0.0595 | 0.3985 | -0.0441 | -0.0015 | **-0.5779** | 0.5017 |
| Q16_1 | No joy in saving | -0.4112 | -0.3775 | 0.1283 | 0.1302 | 0.3039 | 0.5627 |
| Q16_3 | Useless to save | -0.2229 | **-0.7473** | -0.1041 | 0.0435 | 0.0935 | 0.3703 |
| Q16_5 | Saving is good | 0.2700 | **0.7788** | 0.0850 | 0.0034 | -0.0197 | 0.3130 |
| Q17_1 | Important people… | 0.4194 | **0.5328** | 0.1693 | 0.0718 | 0.1203 | 0.4920 |
| Q18_3 | Willing to buy… | 0.2645 | **0.6029** | 0.2407 | 0.0014 | -0.1367 | 0.4899 |
| Q19_1 | Identity -concern | 0.5512 | 0.4269 | 0.1989 | -0.0496 | -0.0299 | 0.4711 |
| Q19_3 | Identity -lifestyle | **0.7162** | 0.1928 | 0.1630 | -0.0387 | 0.1355 | 0.4034 |
| Q20_1 | Cannot relax | -0.0761 | 0.1376 | 0.2118 | -0.0055 | **0.7143** | 0.4202 |
| Q21_1 | Independence | 0.1835 | 0.1752 | **0.6870** | -0.0408 | 0.0045 | 0.4620 |
| Q21_2 | Smart meter | 0.1899 | 0.0637 | **0.7805** | -0.0290 | 0.1101 | 0.3377 |
| Q21_3 | Time-dependent tariff | 0.2616 | 0.1139 | **0.7443** | -0.0768 | 0.0315 | 0.3578 |
| Q21_7 | Feel guilty | **0.7631** | 0.1902 | 0.2363 | 0.0210 | -0.1396 | 0.3058 |
| Q21_8 | Would bother me | **0.7612** | 0.1321 | 0.2615 | 0.0943 | -0.1635 | 0.2991 |
| Q23 | Electricity bill | -0.1847 | 0.1237 | -0.0955 | 0.0433 | **0.6630** | 0.5001 |

*Note:* Figures in bold indicate significant loadings.

**Table A8** Factors used in the regression model, descriptive statistics.

| Variable | Scale items | Mean | Std Deviation |
| --- | --- | --- | --- |
| ***Dependent variable*** |  |  |  |
| The likelihood of accepting battery storage (discrete, 1 to 7) | Extremely unlikely (=1) | 4.7540 | 1.7286 |
|  | Moderately unlikely |  |  |
|  | Slightly likely |  |  |
|  | Neither likely nor unlikely |  |  |
|  | Slightly likely |  |  |
|  | Moderately likely |  |  |
|  | Extremely likely (=7) |  |  |
| ***Independent variables*** |  |  |  |
| *Perceived barriers:* |  |  |  |
| Payback | I don’t think the investment will pay off or the payback period is too long | 2.7828 | 1.3721 |
| Safety | I am concerned about safety | 2.1921 | 1.2584 |
| Technological risk | I am aprehensive of technological change and the risk of not picking the best storage option | 2.4129 | 1.2459 |
| Absence of subsidies | Adequate government subsidies are not available | 2.9605 | 1.4047 |
| *Energy-related motives:* Independence motive | I would like to be more independent from my energy provider. | 4.9803 | 1.3336 |
| Technical interest | I want to have a smart meter. Then I could easily monitor electricity usage. | 4.6804 | 1.5761 |
| Load shifting interest | I want to have a time-dependent electricity tariff. Then I could at least partially transfer my consumption to the cheapest time (e.g. washing at night). | 4.6984 | 1.4966 |
| *Subjective norms* | Most people who are important to me would be happy if I saved electricity | 5.1454 | 1.2661 |
| *Moral emotions* (1) | I would feel guilty if I did not save electricity on a daily basis | 4.9515 | 1.4216 |
| Moral emotions (2) | My conscience would bother me if I did not save electricity on a daily basis | 4.6840 | 1.5151 |
| *Energy-positive attitude* (1) | Saving electricity is useless. | 2.1275 | 1.1829 |
| (2) | Saving electricity is good. | 6.0072 | 1.0213 |
| (3) | I am more willing to buy an appliance with an efficient energy class. | 5.7935 | 1.1717 |
| *Self-identity* (1) | I would be embarrassed not to be seen as having an environmentally friendly lifestyle. | 4.40 | 1.639 |
| *Thermal comfort* (1) | Reduce the use of the air conditioner, by opening the windows, using fans, etc. | 3.7971 | 1.2071 |
| (2) | I find I cannot relax or work well unless the house is airconditioned (in the Summer). | 3.7810 | 1.8079 |
| Electricity bill | What was your electricity bill (estimated) for the last quarter? | 3.2621 | 1.9331 |
| Age | Age of respondent | 4.6894 | 1.6960 |
| Education | Highest level of educational qualifications | 3.5548 | 1.4466 |

**Table A9** Correlation and sampling adequacy test

| Determinant of the correlation matrix | Det = 0.001 |
| --- | --- |
| Bartlett test of sphericity  H0: variables are not intercorrelated | Chi-square = 3719.291 |
|  | Degrees of freedom = 210 |
|  | p-value = 0.000 |
| Kaiser-Meyer-Olkin Measure of Sampling Adequacy | KMO = 0.821 |
